# Needle and syringe sharing practices of injecting drug users participating in an outreach HIV prevention program in Tehran, Iran: A cross-sectional study

**DOI:** 10.1186/1477-7517-2-19

**Published:** 2005-10-07

**Authors:** Mohsen Vazirian, Bijan Nassirimanesh, Saman Zamani, Masako Ono-Kihara, Masahiro Kihara, Shahrzad Mortazavi Ravari, Mohammad Mehdi Gouya

**Affiliations:** 1 Substance Abuse Prevention and Treatment Office, Ministry of Health and Medical Education, Iran; 2 Iranian National Center for Addiction Studies (INCAS), Tehran University of Medical Sciences, Tehran, Iran; 3 Persepolis Society, Tehran, Iran; 4 Department of Global Health and Socio-epidemiology, Kyoto University School of Public Health, Kyoto, Japan; 5 Center for Disease Management, Ministry of Health and Medical Education, Iran

## Abstract

HIV infection rates have reached epidemic proportions amongst injecting drug users (IDUs) in Iran. Although a number of community-based interventions have being implemented in the country, there is little information on the risk behaviors of IDU participants in these programs. This cross-sectional report aimed to compare the risk behaviors of injecting drug users with differential exposure rates to an HIV outreach program in Tehran, Iran. Results indicated that shared use of needle/syringe in the past month was significantly lower among IDUs who received estimated ≥ 7 syringes per week than those who did not [adjusted odds ratio (OR) = 14.36, 95% confidence interval (CI) 2.30–89.56]. While the effectiveness of this outreach program needs further evaluation through a longitudinal investigation, our preliminary findings suggest that the outreach program in Tehran may have been beneficial in reducing direct sharing among those who received more than several needles/syringes from the program.

## Findings

Injecting drug use, to date, has been reported as the main route of HIV transmission in Iran [1,2] and recent figures show that the prevalence of HIV infection has reached to high levels among IDUs in the capital, Tehran [3]. In a series of attempts to prevent HIV transmission among IDUs, an outreach program for HIV prevention was supported by the United Nations Office on Drug and Crimes (UNODC) and the Ministry of Health of Iran in 2003 [4]. As a part of this outreach program, distribution and exchange of needles and syringes have been facilitated by a non-governmental organization (NGO) named Persepolis Society in its single (at that time) drop-in centre and through outreach activities in a neighborhood in Tehran where many drug users live and street-based drug sales have been going on. While methadone maintenance therapy and referrals for detoxification were available at/through the drop-in centre, IDUs who did not want to participate in substitute therapy or detoxification were provided with a package containing 4 syringes/needles, 4 extra needles, water vials, filters, alcohol pads, and 2 condoms at each visit. However, IDUs were provided with large numbers of needles/syringes and condoms if they requested so. Cookers were also offered, but infrequently.

In October 2004, one year after the establishment of the outreach program, a convenience sample of drug users (consisting 213 IDUs and 85 non-IDUs) was recruited at the drop-in centre, and at parks and streets in the area. Active drug users were approached by an ex-user staff member of the NGO for recruitment and were then interviewed by a male researcher not affiliated with the Persepolis Society. In the questionnaire-based interview, participants were asked about their demographics and HIV risk characteristics, as well as their contact with the outreach program, estimated length of contact, and the total number of syringes they received from the program.

Of 131 male injecting drug users who ever received free needles/syringes from the program, data of the 105 male IDUs with a complete set of variables for multivariate analysis were considered for this report and then categorized based on the rate of exposure to the program. The sub-sample was divided into two groups of IDUs who received few needle/syringes (< 7 syringes per week) (37/105 = 35%) and those who received estimated ≥ 7 syringes per week during their participation in the program (68/105 = 65%). While adjusted for demographics and basic drug use characteristics, a logistic regression analysis was performed to assess the association between having received ≥ 7 syringes per week from the program and the related behavioral variables (Table). The research protocol was approved by the Ethical Committee of the Iranian National Center for Addiction Studies at Tehran University of Medical Sciences in Iran and by the Committee for Research on Human Subjects at Kyoto University in Japan.

**Table 1:** Association between having received ≥ 7 syringes/week from the outreach program and related behavioral characteristics

Characteristics	Total No. (%)	Received < 7 syringes per week from the program	Received ≥ 7 syringes per week from the program	Crude odds ratio (95% CI)	*P *value^a^	Adjusted odds ratio (95% CI)	*P *value
Overall	105	37	68	-	-	-	-

Did not share needle/syringe during last month	90 (85.7)	24 (64.9)	66 (97.1)	17.87 (3.75–85.08)	0.000	14.36 (2.30–89.56)	0.004
Did not share a cooker at last injection	37 (35.2)	11 (29.7)	26 (38.2)	1.46 (0.62–3.45)	0.383	0.65 (0.20–2.08)	0.468
Used condom at last sex/never had sex	40 (38.1)	14 (37.8)	26 (38.2)	1.01 (0.44–2.32)	0.968	0.67 (0.22–2.06)	0.488
Know HIV can be transmitted through shared needle/syringe	101 (96.2)	36 (97.3)	65 (95.6)	0.60 (0.06–6.00)	1.000^b^	1.98 (0.14–26.43)	0.605
Ever tested for HIV	63 (60.0)	22 (59.5)	41 (60.3)	1.03 (0.45–2.34)	0.934	0.62 (0.20–1.96)	0.424

Variables shown in this table are controlled for age, ethnicity, marital status, educational levels, recruitment site, length of residency in the area, length of lifetime drug injecting, size of drug use network, and frequency of daily drug injecting.

^a^*P *values based on chi-square test of proportions unless otherwise specified.

^b^Two-tailed Fisher exact test. OR, odds ratio; CI, confidence interval.

The median age of IDUs in this sub-sample was 32.0 years and 67% had educational levels of junior high school or more. Up to 61% were jobless at the time of interview and 52% have ever been married. Among socio-demographic characteristics, only being recruited from the drop-in centre and having lived for 6 years or more in that area were associated with having received ≥ 7 syringes per week from the program in the bivariate analysis.

While 18.9% (7/37) of those who received few needles/syringes from the program reported having injected using a shared needle or syringe at last injection, none of the 68 IDUs who received ≥ 7 syringes per week from the program shared a needle/syringe at last injection (*P *= 0.000) (not in the Table). Also, shared use of needles/syringes in the past month was significantly lower among those IDUs who received ≥ 7 syringes per week than those who did not (adjusted OR = 14.36, 95% CI, 2.30–89.56; *P *value = 0.004). However, there was no difference between two groups in the use of a shared cooker at last injection, condom use during last sex, levels of HIV/AIDS knowledge, and a history of previous HIV testing (Table).

Effectiveness of needle/syringe programs in reducing needle and syringe sharing among IDUs has been shown in many countries [5-9]. Our findings show that those IDUs who received many syringes from the program reported less sharing of needles/syringes in the past month than those who received few syringes. While it is possible that like many other observations [5], the distribution/exchange of free needles/syringes resulted in the lower sharing of needles/syringes among IDUs, a rival hypothesis should also be considered: those IDUs who shared needles/syringes less frequently in the past month may have already had higher risk perception and actively sought and received more syringes from the program. In either situation, the needle and syringe program in Tehran can be considered beneficial in that it either resulted in less sharing of needles and syringes, or at least provided easy access to clean needles/syringes for IDUs whether or not any behavior change occurred.

Cooker sharing that has been less targeted in the program was at high rates among both groups without any significant difference. Because of the importance of cooker sharing in the transmission of blood-borne infections [10,11], the need to address the barriers to distributing cookers has been identified by this study, and efficient ways to prevent cooker sharing are being examined.

This study had several limitations. Though the association between having received many needles/syringes from the program and low rates of sharing needles/syringes in the past month is quite strong, because of the cross-sectional design of this study, it is not possible to draw a conclusion on the direction of this association. In addition, our comparison might have been affected by the selection bias due to convenience sampling. Though yet to be confirmed in studies with more robust design, the above preliminary findings suggest that the HIV outreach program in Tehran may have been beneficial in reducing needle/syringe sharing but not in reducing cooker sharing among IDU participants.

## List of abbreviations

AIDS = Acquired immunodeficiency syndrome 

HIV = human immunodeficiency virus

IDU = injecting drug user

INCAS = Iranian National Center for Addiction Studies

NGO = non-governmental organization

UNODC = United Nations office on Drug and Crimes

## Competing interests

The author(s) declare that they have no competing interests.

## Authors' contributions

MV and BN coordinated the study and collected the data in the field. SZ was responsible for the statistical analysis and writing the paper. MOK and SMR provided assistance in analyzing and interpreting the data. MK and MMG secured funding and supervised the collaborative socio-epidemiologic study between Japan and Iran. All authors read and approved the final manuscript.
